# Limiting Factors in Implementing Pharmacovigilance Principles in the Elderly

**DOI:** 10.7759/cureus.36899

**Published:** 2023-03-30

**Authors:** Heer Shah, Jasleen Nagi, Shivank Khare, Hana Hassan, Anthony Siu

**Affiliations:** 1 General Surgery, Southend University Hospital, Essex, GBR; 2 Internal Medicine, Imperial College London School of Public Health, London, GBR; 3 Internal Medicine, University of Bristol School of Medicine, Bristol, GBR; 4 Internal Medicine, GKT School of Medical Education, London, GBR

**Keywords:** drug-drug interactions, adverse drug events, geriatrics, polypharmacy, pharmacovigilance

## Abstract

The overarching aim of pharmacovigilance is to ensure the safe and effective usage of medication across the population and optimise medicines through holistic considerations. However, within the heterogeneous elderly population, several unique factors are at play, limiting the ability of clinicians to fulfil this aim. A matured physiology influencing the response and effects of drugs, increased polypharmacy enabling drug-drug interactions, and greater consumption of concurrent herbal medicines predispose patients to harmful drug events. This increasingly multimorbid subpopulation requires complex pharmaceutical regimens encouraging inappropriate prescribing and medicine non-adherence leading to suboptimal therapy. Furthermore, restrictive practices in clinical trials commonly exclude elderly patients creating disparities from expected findings within a real-world setting. These issues create an environment where elderly patients are at a heightened risk of adverse drug events and clinicians are forced to make significant decisions from limited information. With projections showing that this demographic will continue growing in size, the true burden of these limiting factors is yet to be realised.

## Introduction and background

The safe and effective use of medicines forms a fundamental part of pharmacovigilance, yet a greater effort is required in ensuring that the primary aims of pharmacovigilance are met in a growing and increasingly heterogeneous elderly population. Worldwide, the proportion of adults above the age of 65 has grown from 4% in 1950 to 9.3% today, representing a shift in demographic patterns and a subsequent increase in the incidence of non-communicable diseases [1]. Published evidence on the increased prevalence of non-communicable diseases with age shows that 64.9% of adults between the ages of 65 and 84 years suffered from at least two chronic conditions, increasing to 80% for those >85 years [2]. Similarly, a study focusing on a population of Medicare beneficiaries found that 67% had multimorbidity, with prevalence increasing with age from 62% for those aged between 65 and 74 years compared to 81.5% at ≥85 years of age [3]. These findings describe a term known as the *third epidemiologic phase of transition* which portrays a population with increased life expectancy, falling mortality rate, and suffering from a greater number of degenerative chronic diseases [4].

Prolonged life expectancy has been attributed to improvements in sanitation, developments in agriculture, increased access to education, as well as a reduction in the number of large-scale wars [5]. However, it is important not to dismiss the significance of pharmacological interventions on longevity through their effect on both treating infectious diseases and improving the quality of life for sufferers of chronic conditions. For example, success in controlling HIV infection through antiretroviral therapy has directly shifted the course of the disease from an acute debilitating illness to a chronic, manageable condition. This has led to notable gains in life expectancy across several African countries [5].

Moreover, a broad econometric study evaluating the impact of new chemical entities (NCEs) found that pharmacological interventions accounted for 40% of the increase in life expectancy between 1986 and 2000 across 52 countries [6]. Despite the clear progress, the increased availability and usage of medications has created an unfortunate cost, realised especially by the elderly population. In this review, we discuss factors that limit our ability to ensure the safe, effective, and efficient use of medicines in the elderly contributing to adverse drug events (ADEs), as well as comment on the struggle to commit to broader pharmacovigilance principles within this demographic.

## Review

Polypharmacy, non-adherence, and alternative therapies

Elderly patients with greater morbidities are likely to be prescribed more medications, thus, contributing to the rise in medicine usage and by extension polypharmacy. Currently, figures show that 70% of the population is prescribed one or more medications, with this number rising to 90% in patients >65 years of age. Similarly, the proportion of adults receiving five or more medications has increased considerably over the last decade [7]. It is worth noting that treatment for chronic conditions is often long-term and in many cases life-long [8]. Therefore, a pattern emerges of an ageing and increasingly morbid population requiring multiple simultaneous prescriptions of prolonged duration.

On its own, polypharmacy poses difficulty when implementing pharmacovigilance principles. It disrupts our ability to ensure the safe use of numerous concurrent medications creating complex risk-benefit analyses that may lead to suboptimal care. Moreover, adverse events stemming from polypharmacy highlight the gaps in our knowledge of harmful drug-drug and drug-disease interactions. A cross-sectional population study found that the prevalence of polypharmacy (defined as the concomitant use of five or more medicines) was 32% in adults >60 years [9]. Similarly, a US survey found that 90% of patients >65 years old had taken at least one medication in the preceding week, and from those sampled, 12% had taken ≥10 medications within the same period [10]. This represents a disproportionate consumption of medicines by the elderly in relation to the number of adults within this demographic [11]. Additionally, polypharmacy will play an increasingly large part in elderly medicine as the elderly population follows its forecasted growth and, thus, poses a hurdle in achieving optimal care.

Numerous studies have highlighted that polypharmacy is an independent risk factor for ADEs, with 60% stemming from drug-drug interactions [12,13]. Furthermore, the risk of ADEs increases exponentially, with each subsequent medication growing from 13% for patients taking two medications to 82% for patients taking more than seven medications [14]. Specifically, polypharmacy can aggravate the risk of harmful cytochrome P450 (CYP) drug-drug interactions. CYP isozymes play a key role in the metabolism of several drugs, including paracetamol, haloperidol, and warfarin, which are commonly prescribed to the elderly. However, numerous drugs can induce or inhibit these isozymes and, subsequently, influence the bioavailability of a second drug. Therefore, reactions with isozymes can result in harmful adverse events or a reduction in the therapeutic effect of these common drugs [15]. Equally, another study found that 80% of their elderly patient sample who took five or more medications had at least one CYP-related drug-drug interaction [16]. In 19% of cases, harmful interactions necessitated immediate drug adjustment, and 39% of patients required prolonged follow-up, representing both a patient and broader service cost. With increased polypharmacy, the number of simultaneous drugs competing for the same isozyme will increase, which may result in novel downstream effects with the potential to cause harm [16]. Furthermore, as pharmaceutical regimens become broader and increasingly complex, the ability to predict ADEs decreases, representing a significant limiting factor in our pharmacovigilance attempts. Therefore, polypharmacy imposes particular caution towards clinicians deciding to introduce additional medications.

A further consequence of polypharmacy and complex medicine regimens is non-adherence to prescribed medicines or consumption of medications outside of their intended regimen [17,18]. Figures for levels of non-compliance range between 20% and 40% but are associated with significant monetary and human costs [19,20]. Subtherapeutic levels of medication resulting from non-compliance can facilitate adverse events and may exacerbate underlying disease processes. For example, a study showed that non-compliance to bisphosphonates contributed to a significantly increased risk of hip fractures in patients with osteoporosis [21]. Similarly, a large study evaluating antihypertensive compliance in patients with high blood pressure found that poor compliance correlated with poor blood pressure control and increased levels of hospitalisations [22]. Difficulty conveying prescription regimens along with a lack of understanding of why specific medicines are prescribed represent limitations in our broader pharmacovigilance attempts to effectively educate elderly patients.

Drug labels are often unclear and difficult to understand, thus, impacting adherence to medication [23]. A 2008 study asking participants to interpret 10 common prescription bottles with instructions on use demonstrated that 78% of them misunderstood one or more instructions [24]. This is particularly distressing as polypharmacy warrants a clear understanding of instructions and warnings to appropriately navigate complex pharmaceutical regimens. Elderly patients are especially implicated as age-related slowing of cognitive processes may exaggerate the difficulty in interpreting instructions clearly. Studies have provided evidence of this correlation noting poorer compliance with increasing age and attributing this finding to progressive functional decline [18]. A further limiting factor concerning compliance is the formulation and packaging of medicines. Child-proof lids, for example, may prove difficult for elderly patients with impaired strength, reduced dexterity, or those with musculoskeletal disorders such as arthritis. Similarly, certain formulations may be inappropriate for patients with dysphagia or visual impairments [24]. Increased inclusion in treatment decisions, education on the purpose of specific prescribed medications, and elderly-friendly modifications to the formulation and packaging of medicines may help reduce non-compliance and associated ADEs.

Additionally, there is growing evidence that the elderly are high consumers of alternative and herbal therapies alongside prescribed medications. Evidence from a large cross-sectional study of elderly Turkish individuals reported that 72% used herbal products and prescribed medicine(s) concurrently. Of the herbal products identified, *nettle* was commonly used by participants. This is concerning as nettle has established links to gastric irritation and can potentiate the effect of diuretics [25]. Similarly, a German study found that 61% of participants consumed some form of alternative or herbal medication which was seldom on the advice of their pharmacists or doctors. While the majority of supplements from this sample presented no severe risk, 3% of cases were associated with a significant risk to the individual when used in combination with their prescribed medicines [26]. While most supplement-drug interactions posed no substantial harm, 5% would pose serious concern and require intervention [27]. Furthermore, studies have shown that elderly patients often consume multiple herbal or alternative medications simultaneously, thus, exponentially increasing the risk of both adverse drug-supplement and adverse supplement-supplement interactions [25,28].

As clinical trials seldom specifically focus on the use of herbal and supplementary medicines, it is difficult to establish a safety profile for these medicines. Therefore, adverse events related to the consumption of these supplementary medicines or from interactions with prescribed medications may not be easily recognised. Moreover, herbal medications are commonly missed from a drug history with reports of between 25% and 50% of patients not disclosing their herbal medicine consumption to their general physician [26,29]. The lack of knowledge surrounding the impact of these supplements and their prevalence within the elderly population represents a limiting factor in our ability to safely prescribe medications without the risk of harmful interactions. Trends showing greater use of herbal medications in the elderly also represent a shortfall in our pharmacovigilance efforts to better educate the public about the effects of available supplements. A greater effort is required in educating individuals that cheap and easily accessible natural herbal remedies do not come risk-free.

Physiological changes with age, inappropriate prescribing, and adverse drug reactions

In addition to the difficulty of optimally prescribing medications that provide therapeutic benefits without prompting harmful drug-drug interactions, elderly people are also more prone to ADEs akin to age-related changes to their physiology. The increase in the proportion of adipose tissue with age is well documented but can have profound effects on lipid-soluble drugs such as benzodiazepines. These drugs are subsequently more widely distributed throughout the body leading to a sustained effect and prolonged elimination [30]. The kidneys are also subject to specific age-related changes. Structural changes accompanied by a decrease in the number of glomeruli contribute to the reduction in renal clearance [31,32]. These changes influence the excretion of numerous drugs, including digoxin, prompting dose reduction to reduce potential harm [33]. Pharmacodynamic changes including changes to end organ receptor sensitivity can exaggerate the response to certain drugs such as analgesics, but, similarly, a reduction in the number of receptors can diminish the response to other drugs such as beta-blockers [34]. Therefore, a paradoxical situation exists where the elderly population have an increased predisposition to ADEs but is also the most likely recipient of multiple medications.

The lack of knowledge surrounding changes in pharmacokinetic and pharmacodynamic processes with age leaves clinicians commonly prescribing inappropriate medications at suboptimal doses. By extension, the prevalence of inappropriate prescribing among the elderly is high, with one study reporting that up to 20% of elderly patients were affected. Common inappropriate prescriptions included long-acting benzodiazepines, nitrofurantoin, and non-steroidal anti-inflammatory drugs (NSAIDs) in patients with a history of duodenal ulcers [35]. A contributing factor stems from our increasingly specialised and segregated clinical practice which leaves elderly patients with a range of comorbidities experiencing multiple prescribers, each focusing on attenuating their particular disease interest. Although the provision of specialised care allows clinicians to best utilise their expertise, the disruption in continuity of care and lack of holistic considerations contribute significantly to ADEs. A study of 405 patients supported this view and found that the number of prescribing physicians was an independent risk factor for self-reporting ADEs [36]. Additionally, medication discrepancies during hospital admission are common and may be attributed to the inability to obtain a clear drug history, a consequence of multiple prescribers, or fatigue from prescribing a large number of medications. A Canadian study demonstrated that 40% of patients >70 years of age admitted to elderly care units were subject to medication discrepancies. The study also found that a substantial proportion of these prescription-medication discrepancies were associated with potential harm [37].

Efforts to reduce inappropriate prescribing in the elderly include medicine reconciliation where the purpose for each medication is defined probing decisions on its appropriateness. Medical reviews by geriatricians or by doctors with specialist interests in the elderly also help guide medicine optimisation and reduce the risk of ADEs [38]. However, optimal care for elderly patients should not be the sole responsibility of geriatricians, especially because the vast majority of specialties will house elderly patients. Therefore, efforts to improve holistic care for the elderly, optimise medications, and implement the values of pharmacovigilance should be a shared responsibility among all clinicians who may encounter elderly patients. Guidelines from the National Service Framework for older people state that medication reviews should be conducted on an annual basis in those aged over 75 years and biannually in those taking more than four medications [39]. Such reviews prompt dose adjustments and appropriate medicine discontinuations.

However, implicit measures such as medication reviews and reconciliations that require clinical judgement are time-consuming and subjective. By contrast, explicit measures that review prescribing appropriateness do not require exhaustive clinical judgement [40]. One common screening tool that uses explicit measures is Beers criteria. While widely cited in the literature, Beers criteria appear more relevant to US prescribing patterns and US drug availability, thus limiting its transferability [41]. Recognising these shortcomings, newer explicit screening criteria have been developed with improved applicability to the NHS. For example, the STOPP tool which stands for the Screening Tool of Older Persons’ Prescriptions comprises 65 clinically significant indicators for potentially inappropriate prescribing [42]. In a primary care setting, the STOPP tool correctly identified 284 (21.4%) potentially harmful medication prescriptions [43]. Other methods of reducing inappropriate medications incorporate the use of electronic prescribing which can send notifications to report these events in real time. Nevertheless, reliance on automated systems does not replace sound clinical acumen, and clinicians should be encouraged to improve their knowledge and treatment of elderly patients.

Lastly, a separate aim of pharmacovigilance involves the cost-effective use of medicines [44]. The cost of inappropriate prescribing presents an opportunity cost where money could be better spent elsewhere. Although no monetary estimates for the true cost of inappropriate prescribing currently exist, studies have reported that drug wastage in primary care costs the NHS approximately £100 million, an average of £10 per person above the age of 65 [45]. Optimising medications holistically for elderly patients will foster a more efficient and effective practice and should be practised at all levels of care.

Moreover, increasing education on the physiological changes with age and pharmacological vulnerabilities in the elderly will foster a greater awareness of the safe, effective, and rational use of medicines. However, a focus on also improving the recognition of adverse drug reactions (ADRs) in the elderly should be included as part of a comprehensive pharmacovigilance programme. The presentation of ADRs in the elderly is often atypical and can imitate underlying disease processes such as global decline akin to frailty and dementia [32]. Furthermore, ADRs can also present as vague symptoms, including constipation, hallucinations, tremor, and falls, which are mistakenly attributed as primary diagnoses rather than secondary to medication [31,46]. The difficulty in recognising true ADRs creates a cycle where clinicians are often persuaded to treat vague symptoms with additional medications instead of accurately identifying and removing the culprit drug. A clinical example of this loop exists through the use of NSAIDs which are prone to causing reflux and gastric irritation. Instead of replacing the NSAID with a suitable alternative, clinicians opt to prescribe a proton-pump inhibitor to treat the gastrointestinal upset but, subsequently, cause the iatrogenic ADE of diarrhoea [33]. This concept is known as a *prescription cascade* contributing significantly to polypharmacy within the elderly and increasing the likelihood of both drug-drug interactions and subsequent ADRs. The underrecognition of ADRs stands as a key limiting factor in our ability to use drugs effectively in the elderly and promotes further unnecessary prescriptions that may cause iatrogenic harm to the patient. Encouraging clinicians to include ADRs within their differential diagnosis and providing education on the nature of ADRs in the elderly will foster better and more optimal use of medicines in line with the aims of pharmacovigilance.

Underrepresentation in clinical trials

Post-market surveillance provides real-world evidence on notable ADEs concerning the elderly and increases our understanding of specific susceptibilities. It is a form of reactive pharmacovigilance where adverse events occur, causality from the implicated drug is determined, and future ADEs are prevented. Unfortunately, this process is time-consuming, prone to subjectivity, and results in considerable suffering as a consequence of the ADE. Greater evidence stemming from randomised controlled trials (RCTs) which specifically evaluate drugs in the elderly will reduce reliance on post-market surveillance for ADEs and foster appropriate medicine use from improved knowledge about medicines. Data from RCTs will help produce safe, effective, and translatable therapies promoting a proactive form of pharmacovigilance. Ideally, trials should be populated with patients of similar demographics and morbidities to those that will likely receive the intervention within the real-world setting. This would allow thorough testing of the safety and efficacy of drugs before general use and flag any unwarranted effects at a pre-market stage, thus reducing harm. The International Conference on Harmonization of technical requirements for the registration of pharmaceuticals for human use specified that a study population should be representative of the population that will consume the drug and that studies should include a minimum number of elderly patients [47]. Despite such guidance, the omission of elderly patients from clinical trials remains common, creating unrepresentative research populations and proving to be a limiting factor in our ability to safely prescribe effective and appropriate medications.

Numerous studies have highlighted the lack of evidence concerning the efficacy and safety of drugs in the elderly population. A study evaluating drug approval documents within the Food and Drug Administration database found that over half had no reports on the safety or efficacy in the elderly, and over one-third had no reports on specific pharmacokinetic analyses [47]. Furthermore, a systematic review highlighting the characteristics of RCTs found that only 2% of all RCTs in 2012 were designed for the elderly, representing a significant gap in our knowledge of drug effects in this demographic [48]. A study concerning four commonly prescribed drugs to the elderly, namely, pioglitazone, rosuvastatin, risedronate, and valsartan, showed that the proportion of patients within the studies above the age of 65 years was significantly lower than the comparable proportion within clinical practice. This particularly highlights the view that elderly patients are disproportionately represented in RCTs of drugs that they are likely to be recipients of [49].

Additionally, studies focusing on conditions with higher prevalence in the elderly population also fall short of hosting representative study populations and, therefore, negate the validity of results and reduce the ability to extrapolate results into older demographics. For example, the prevalence of Parkinson’s disease (PD) increases from 0.6% for those >60 years to 3.5% for those >85 years [50]. However, a systematic analysis assessing the frequency of exclusion of older patients in PD research found that 49% of studies imposed an upper age limit of exclusion (mean age: 79.3 years) [51]. Similarly, 25% of trials investigating treatments for heart failure excluded patients based on an arbitrary age limit, despite heart failure being the most common cause of hospital admissions among elderly patients [52]. In addition to the arbitrary age limit, other poorly justified exclusion criteria were common, including non-specific comorbidities, use of concurrent medications that would not impact the study protocol, and visual impairments that would not provoke specific safety concerns [53].

Other reasons for exclusion include poorer compliance in the elderly population and, therefore, trouble adhering to the trial protocol, increased risk of drug interactions due to polypharmacy, and cognitive challenges which hinder consent for inclusion in trials [54]. Furthermore, there may be logistical issues including transport to both the study site and for follow-up visits and a tendency towards higher dropout rates from studies due to illness or death during the trial. Lastly, due to the heterogeneity among older patients, sponsors may voice concerns that the treatment effect will be diluted leading to non-significant results which may further dissuade their inclusion [55]. Nevertheless, the elderly population are entitled to effective evidence-based treatments, but the restrictive eligibility present in trials and subsequent underrepresentation continues to diminish the validity of results and cloud the true therapeutic value of interventions. Therefore, clinicians must rely on evidence from a younger, healthier population compromising their ability to tailor treatments to a population with different physiology. A significant consequence is that unrepresentative studies have low power and may not be able to detect significant ADRs in the elderly population. A large RCT highlighting this showed evidence that the use of spironolactone significantly reduced the risk of death by 30% in patients with heart failure [56]. However, after this publication and likely due to the increased use of spironolactone by clinicians, reports of morbidity and mortality associated with hyperkalaemia became increasingly frequent [57]. Possible reasons for not foreseeing this outcome likely stem from the restrictive eligibility criteria of the original study. Real-world patients were likely older, had reduced kidney function, and had concurrent prescriptions of beta-blockers that may have contributed to the exacerbation of hyperkalaemia [58]. Including elderly patients in clinical trials and performing clinical trials specific to this demographic will increase our confidence in prescribing evidence-based appropriate treatments and develop our understanding of factors that contribute to ADEs.

## Conclusions

Drugs are developed, optimised, and delivered with the average person in mind leaving the prerequisites of an elderly population commonly neglected despite a disproportionate consumption of medication by this age group. Furthermore, the growing elderly subpopulation often suffers multiple morbidities, necessitating complex pharmaceutical regimens which increase the likelihood of harmful drug-drug interactions and suboptimal therapy from non-adherence. Lack of evidence stemming from narrow trial populations coupled with inadequate expertise on the physiological changes with age prompt inappropriate prescribing of medicines, delays in detecting ADEs, and iatrogenic harm. Moreover, homogeneously applying age cut-offs to deny treatment instead of holistically evaluating elderly patients in terms of their frailty and thus suitability for treatment highlights a gross lack of understanding regarding this demographic and promotes an impersonalised approach to treatment.

Drug use in the elderly along with the characteristics of a heterogenous elderly population must be better understood for us to extend our safe and effective use of medicines to this subpopulation. While greater inclusion of elderly patients in trials has helped improve evidence on the benefits versus harms of medicines in this age group, arbitrary age limits continue to be imposed, thus ignoring very old patients. Therefore, a significant challenge will be to consistently have representative clinical trials producing better quality research and safely incorporating these findings into complex management plans. The projected growth of the elderly demographic paints an upward spiral for polypharmacy, suboptimal therapy, and increased mortality as a result of ADEs. Thus, we must not only recognise current limiting factors but also create actionable Initiatives concerning multiple parties to achieve safer and more effective therapies for our future elderly population.
